# Supplementary material for: Identification of photoperiod-induced specific miRNAs in the adrenal glands of Sunite sheep (Ovis aries)
Source: Front Vet Sci. 2022 Jul 22;9:888207. doi: 10.3389/fvets.2022.888207 (PMC9354845; doi:10.3389/fvets.2022.888207)
Supplement: Supplementary file 1 [file Data_Sheet_1.ZIP › Supplementary file/miRDeep2 file.zip!/miRDeep2 file/Novel_Structure/SPLP42A2/Novel_411.pdf]

Provisional ID	:	NC_019464.2_17545
Score total	:	0.7
Score for star read(s)	:	-1.3
Score for read counts	:	0
Score for mfe	:	0.4
Score for randfold	:	1.6
Score for cons. seed	:	
Total read count	:	44
Mature read count	:	44
Loop read count	:	0
Star read count	:	0

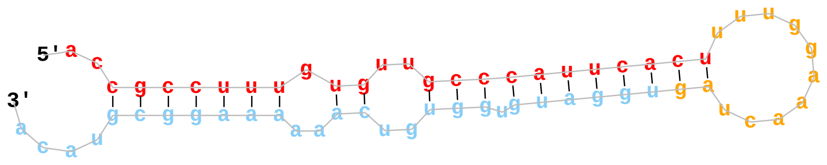

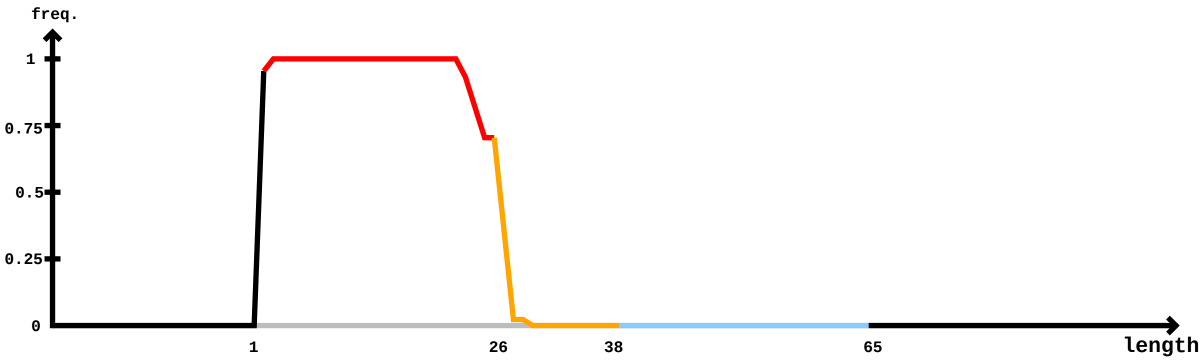

Mature Star

```
5'- auuccuuaugcgguuuucuuaccgccuuuguguugcccauucacuuuuggaaacuaguggauguggugucaaaaaaggcguacacaaaacacuuucgcagcuuuuuuuccugccc   -3'   exp
    ............(((((.....(((((((.((..(((((((((((..........)))))))).)))..))..)))))))).....)))))......((((.........))))..    reads      mm        sample
    ....................accgccuuuguguugcccauu..........................................................................    3          0         seq
    ....................accgccuuuguguugcccauuc.........................................................................    5          0         seq
    ....................accgccuuuguguugcccauuca........................................................................    5          0         seq
    ....................accgccuuuguguugcccauucacu......................................................................    21         0         seq
    ....................accgccuuuguguugcccauucacuu.....................................................................    7          0         seq
    ....................accgccuuuguguugcccauucacuuuu...................................................................    1          0         seq
    .....................ccgccuuuguguugcccauucacu......................................................................    2          0         seq
```
